# *Bacillus coagulans* LMG S-24828 Impairs *Candida* Virulence and Protects Vaginal Epithelial Cells against *Candida* Infection In Vitro

**DOI:** 10.3390/microorganisms12081634

**Published:** 2024-08-10

**Authors:** Luca Spaggiari, Andrea Ardizzoni, Natalia Pedretti, Ramona Iseppi, Carla Sabia, Rosario Russo, Samyr Kenno, Francesco De Seta, Eva Pericolini

**Affiliations:** 1 Clinical and Experimental Medicine PhD Program, University of Modena and Reggio Emilia, 41125 Modena, Italy; luca.spaggiari@unimore.it; 2 Department of Surgical, Medical, Dental and Morphological Sciences with Interest in Transplant, Oncological and Regenerative Medicine, University of Modena and Reggio Emilia, 41124 Modena, Italy; andrea.ardizzoni@unimore.it (A.A.); samyr.kenno@unimore.it (S.K.); 3 Department of Medical Sciences, University of Trieste, 34129 Trieste, Italy; natalia.pedretti@units.it; 4 Department of Life Sciences, University of Modena and Reggio Emilia, 41125 Modena, Italy; ramona.iseppi@unimore.it (R.I.); carla.sabia@unimore.it (C.S.); 5 Giellepi S.p.A., Via G. Verdi, 41/Q, 20831 Seregno, Italy; rosario.russo@giellepi.it; 6 Department of Obstetrics and Gynecology, IRCCS San Raffaele Scientific Institute, University Vita and Salute, 20132 Milan, Italy; fradeseta@gmail.com

**Keywords:** *Bacillus coagulans*, probiotics, *Candida*, vaginal epithelial cells

## Abstract

Probiotics are living microbes that provide benefits to the host. The growing data on health promotion, following probiotics administration, increased interest among researchers and pharmaceutical companies. Infections of the lower genital tract in females, caused by a wide range of pathogens, represent one of the main areas for the use of probiotics and postbiotics. Vulvovaginal candidiasis (VVC) affects 75% of women of reproductive age at least once during their lifetime, with 5–8% developing the recurrent form (RVVC). The disease is triggered by the overgrowth of *Candida* on the vaginal mucosa. Here, in order to establish its probiotic potential in the context of VVC, we evaluated the anti-fungal effects of the spore-producing *Bacillus coagulans* LMG S-24828 against *C. albicans* and *C. parapsilosis* as well as its beneficial effects in counteracting *Candida* vaginal infection in vitro. Our results show that both live *B. coagulans* and its Cell-Free Supernatant (CFS) exerted antifungal activity against both fungi. Moreover, live *B. coagulans* reduced hyphal formation, inhibited *C. albicans* adhesion to vaginal epithelial cells, showed co-aggregation capacity, and exerted a protective effect on vaginal epithelial cells infected with *C. albicans*. These data suggest that *B. coagulans* LMG S-24828 may provide benefits in the context of *Candida* vaginal infections.

## 1. Introduction

Vulvovaginal candidiasis (VVC) is a widespread disease among the female population. Three-fourths of women worldwide are estimated to be affected by VVC at least once during their reproductive age. Moreover, 5–8% of them develop the recurrent form (RVVC), consisting of more than four episodes of VVC per year. Both VVC and RVVC have a significant impact on the life quality and mental wellbeing of the approximately 138 million affected women [1,2]. VVC is a symptomatic inflammation of the vagina, and it is mainly caused by *Candida albicans* (*C. albicans*). Infections due to other species of the genus *Candida*, such as *C. parapsilosis*, *C. glabrata*, *C. tropicalis*, and *C. krusei*, are less common, but they are more frequently associated with the recurrent form. VVC symptoms, which include itching, burning, discharge, and dyspareunia, are caused by a hyperinflammatory response to *Candida* rather than by the presence of the fungus, which normally dwells in the vagina as a commensal [3]. As a colonizer of the healthy vaginal niches, *Candida* dwells on the vaginal mucosa in the form of yeast, and its proliferation and pathogenicity are constantly inhibited by the immune system and by resident bacteria, such as *Lactobacillus* spp. The pH of a normal vaginal environment typically ranges between 3.5 and 4.5, because of the lactic acid produced by *Lactobacillus* spp. [4,5]. Alterations in microbial composition and immune system dysfunctions may increase the risk of *Candida* infection. Pregnancy, diabetes mellitus, hormones administered as oral contraceptives, wide-spectrum antibiotic therapy, and genetic predisposition are among the main VVC predisposing factors [6]. The VVC onset is linked to *C. albicans* morphological transition from yeast to hyphal form. The subsequent epithelial invasion and cell damage trigger an intense immune and inflammatory response; the latter ultimately causes the symptoms of the disease.

VVC episodes are typically treated with oral or topical azole antifungals (mainly fluconazole). However, despite the therapy effectively granting relief from symptoms, in about 50% of cases, reinfection occurs after 6 months. For RVVC, elective therapy requires continuous treatment with fluconazole for 6 months. Nevertheless, more than half of the women show symptom relapse a few months after the end of fluconazole treatment. Overall, azole-based treatment has a limited long-term efficacy and consequently high costs [7,8]. Moreover, the occurrence of clinical isolates resistant to azoles further indicates the need to define alternative therapeutic approaches [9].

In this regard, interest in probiotics and their role in human health has increased in recent years, because of their excellent performance in preventing and treating several diseases and the increasing demand for natural medicines by consumers [10]. Probiotics are living microorganisms that, when consumed in appropriate quantities, provide health benefits to the host, as defined by the World Health Organization. Antimicrobial activity against pathogens, modulation of the immune system, and enhancement of essential nutrient bioavailability are among the major benefits granted by probiotics consumption. Probiotics have also been demonstrated to interact with the host microbiota. The latter may influence several organs, typically by immunomodulatory signals. Indeed, connections between the gut microbiota and the brain, liver, lung, skin, and vagina have been already demonstrated [11,12]. Probiotics are also being studied in psychiatry as a novel therapeutic approach aimed to treat conditions such as mood disorders, anxiety, and depression [13,14].

Probiotics are typically Gram-positive bacteria, mainly belonging to the genera *Lactobacillus* and *Bifidobacterium*. As recently reviewed, probiotics are able to form a barrier on the vaginal epithelium that prevents *Candida* adhesion both directly (through competition for the epithelial binding sites) and indirectly (through mechanisms of coaggregation). In addition, probiotics compete with *Candida* for nutrients and inhibit the fungal dimorphic transition, as well as secretion of virulence factors, such as aspartyl proteases (Sap) and the expression of hyphal-associated virulence factors. Interestingly, probiotics have also been demonstrated to potentiate epithelial response to *Candida*, through reinforcement of epithelial tight junctions [3]. Recently, other genera have been demonstrated to show probiotic activity [15,16]. Spore-producing bacteria, such as several species belonging to the genus *Bacillus*, have been frequently employed in commercial formulations due to their properties. Indeed, they can survive various industrial processes, and they remain stable at room temperature over non-sporulating probiotic species [17]. Most probiotic formulations are taken orally. In spore-based formulation probiotics, spore germination and bacterial proliferation occur on the gut mucosa surface. 

Evidence of the benefits of probiotics in VVC treatment or prevention is yet to be fully demonstrated, but preclinical studies and clinical trials suggest a beneficial role of several probiotics against *Candida* vaginal infections [18,19]. Although there is no sufficient evidence to support the replacement of antifungals with probiotics to date, the latter represent an interesting approach at least to preventing the recurrent forms and an aid to restoring vaginal eubiosis [20,21].

The treatment of VVC with antifungal agents consists mainly of intravaginal and sometimes of oral administration of fluconazole and ketoconazole. In the context of bacterial vaginosis, it has already been demonstrated that oral or intravaginal administration of different species of *Lactobacillus* increases the number of vaginal lactobacilli, which in turn may provide a mechanical barrier against *Gardnerella vaginalis*, therefore preventing the pathogen’s adhesion to the vaginal epithelium [22]. Concerning VVC, there is at least one probiotic strain, *Saccharomyces cerevisiae* CNCM I-3856, already available on the market, that is provided as an oral formulation, showing the capacity to migrate from the intestine to the vagina [23,24]. Other probiotic strains, such as *L. plantarum* P17630, analyzed in clinical trials, show the capacity to colonize the intestine and the vaginal cavity, providing benefits against VVC [25].

Here, by an in vitro study, we assessed if the *Bacillus coagulans* LMG S-24828 strain (recently reclassified as *Heyndrickxia coagulans* and marketed as Weizy^®^ by Giellepi S.p.A.) could exert anti-*Candida* activity in order to establish its probiotic potential in the context of VVC. The results presented here demonstrate that *Bacillus coagulans* LMG S-24828 counteracts *Candida* virulence and exerts a beneficial effect against vaginal epithelial cell infection by *C. albicans.* Notwithstanding the obvious limitations of an in vitro study such as the present one, these promising preliminary data suggest that this bacterial strain has all the potentialities to be employed as a spore-forming probiotic for counteracting *Candida* spp. vaginal infections. In addition, the possibility of oral administration, made possible by the spore germination capacity on the intestinal epithelium, makes it a very interesting potential product to be employed for the prevention/treatment of VVC.

## 2. Materials and Methods

### 2.1. Microbial Strains and Growth Conditions

The strain of *Bacillus coagulans* LMG S-24828 (Weizy^®^) was provided by Giellepi S.p.A (Milan, Italy) in the form of lyophilized spores. Spores were stored at 4 °C and germination was achieved by incubation in Tryptic Soy Broth (TSB) (Condalab, Madrid, Spain) for 24 h at 37 °C, under agitation. Bacteria, obtained from spores’ germination, were used in their exponential growth phase for each experiment. The reference strains *Candida albicans* SC5314 (ATCC MYA-2876) and *Candida parapsilosis* CLIB214 (ATCC 22019) were employed. Both strains had been stored in frozen stocks at −80 °C in Sabouraud Dextrose Broth (SDB) (Condalab, Madrid, Spain) supplemented with 15% glycerol. After thawing, the fungi were grown in Yeast Extract–Peptone–Dextrose broth (YPD) (Scharlab S.L., Sentmenat, Spain) and incubated at 37 °C under aerobic conditions for 24 h. Fungi in the exponential growth phase were used in the experiments.

### 2.2. pH Measurement of Microbial Culture

*Bacillus coagulans* (5 × 10^6^ CFU/mL) and *C. albicans* or *C. parapsilosis* (both at 5 × 10^3^ CFU/mL) were added to a medium consisting of 5 mL of YPD broth and 5 mL of TSB. The co-culture was incubated at 37 °C for 24 h, under agitation. Samples containing only the bacterium, only the fungus, or sterile media were also included as controls. After incubation, samples were centrifuged at 4000 rpm for 15 min and sterilized by filtration; then, pH was measured using a pHmeter (Hanna Instruments, Villafranca, Italy).

### 2.3. Analysis of Bacillus coagulans Effect on Candida Growth 

The impact of *B. coagulans* on *C. albicans* and *C. parapsilosis* growth was evaluated by incubating the fungi with or without the bacterium. A total of 500 µL of *B. coagulans* (5 × 10^6^ CFU/mL) in TSB was seeded in a 24-well plate with 500 µL of *C. albicans* or *C. parapsilosis* (both at 5 × 10^3^ CFU/mL) in YPD broth. As a control sample, both *Candida* species were incubated with sterile TSB without *B. coagulans*. To assess acidification contribution, 500 µL of both fungi was seeded with 500 µL of TSB that had been acidified with chloridric acid (Mallinckrodt Baker, Webster Groves, MO, USA) to pH 4.4. The plate was incubated at 37 °C for 24 h. Then, culture supernatants were collected together with adherent fungi, which were detached by washing the wells with Soybean-Casein-Digest-Lecithin-Polysorbate-80 broth (SCDLP80, Biotec, Dueville, Italy). The *Candida* burden was quantified by serially diluting the samples and seeding them on Sabouraud Dextrose Agar (SDA) (Condalab, Madrid, Spain) supplemented with chloramphenicol (50 mg/L) (Biolife Italiana Srl, Monza, Italy). Colony Forming Units (CFU) were counted after 24–48 h of incubation at 37 °C.

### 2.4. Preparation of Cell-Free Supernatant (CFS) from Bacillus coagulans and Its Effect on Candida Growth

The *B. coagulans* broth culture obtained from spore germination, as detailed above, was centrifuged at 4000 rpm at 4 °C for 15 min. Then, the supernatant was discarded, and the pellet was resuspended in 1 mL of TSB. Optical density (OD) at the wavelength of 595 nm was measured using a spectrophotometer (Sunrise, Tecan, Männedorf, Switzerland), and bacterial concentration was calculated from the OD values by means of a stored standard curve. Then, 5 mL of TSB containing *B. coagulans* (1 × 10^8^ CFU/mL) was incubated at 37 °C for 24 h, under agitation. At the end of the incubation, CFS was obtained by centrifugation of the bacterial culture at 4000 rpm at 4 °C for 15 min, as previously described [26]. Briefly, the supernatants were collected, filter-sterilized with 0.22 µm syringe filters (Corning Incorporated, Corning, NY, USA), and stored at −80 °C until their use. The sterility of CFS was confirmed by incubating an aliquot of each CFS at 37 °C for 72 h and then seeding it on Tryptic Soy Agar (TSA) (Condalab, Madrid, Spain). The effect of CFS on *C. albicans* and *C. parapsilosis* growth was evaluated by seeding 100 µL of YPD containing *C. albicans* or *C. parapsilosis* (1 × 10^4^ CFU/mL) in a 96-well plate, together with 100 µL of CFS (or the same volume of TSB in the control samples). The plate was incubated at 37 °C and the cultures’ OD values were kinetically measured (reading cycle: 2 h) for 18 h at the wavelength of 570 nm by a spectrophotometer (Sunrise, Tecan, Switzerland).

### 2.5. Bacillus coagulans Impact on C. albicans Hyphae Formation

A filamentation assay was performed on chamber slides (Nunc Lab-Tek II, Thermo Fisher Scientific, Waltham, MA, USA). A total of 100 µL of *C. albicans* in YPD broth supplemented with 10% of fetal bovine serum (FBS) (SIAL Srl, Roma, Italy) was seeded in the well of the chamber slide along with 100 µL of *B. coagulans* in TSB supplemented with 10% FBS. As a control sample, the fungus was incubated with sterile TSB supplemented with 10% FBS. The chamber slide was placed at 37 °C with 5% CO_2_ for 4 h. Fifteen minutes before the end of the incubation, 40 µL of 1% Uvitex 2B fluorescent dye (Polysciences, Inc., Warrington, PA, USA) was added to each well. After the end of incubation, wells were gently washed twice with 200 µL of phosphate buffered saline (PBS) (SIAL Srl, Roma, Italy) that had been warmed at 37 °C. Then, samples were fixed with 1% paraformaldehyde (200 µL/well, 30 min of incubation at 4 °C). Finally, wells were washed twice with 200 µL of cold PBS (4 °C) and they were allowed to dry. The chamber slide was then disassembled by removing the plastic frame that kept the wells isolated, and the slide surface was treated with ProLong Gold Antifade Reagent (Molecular Probes, Invitrogen, St. Louis, MO, USA). The fluorescence of fungi was visualized by epifluorescence microscope Nikon Eclypse 90i (Nikon Instruments, Tokyo, Japan). Yeast cells and hyphal fragments were counted (at least three fields for each experimental condition) and the percentage of hyphal fragments in each sample was calculated.

### 2.6. Evaluation of Bacillus coagulans Ability to Co-Aggregate with C. albicans

To establish if *B. coagulans* was able to co-aggregate with *C. albicans*, 50 µL of yeasts resuspended in PBS (2 × 10^7^ CFU/mL) and 50 µL of *B. coagulans* suspension in PBS (2 × 10^9^ CFU/mL) were seeded in a U-bottom 96-well plate (Corning Incorporated, Corning, NY, USA) and incubated under gentle agitation at 37 °C for 1 h. Samples containing the bacteria or the fungus alone were also included as controls. Co-aggregation rate was visually evaluated and scores from 0 to 4 were assigned, according to a previous report [19]. Briefly, a score of 0 means no aggregation; a score of 1 is characterized by aggregates with small clusters; a score of 2 is characterized by aggregates with larger numbers of yeasts; a score of 3 includes clumps visible with the naked eye, containing large numbers of yeast cells; a score of 4, the maximum, is assigned when large clumps become visible with the naked eye at the center of the well.

### 2.7. Long-Term Effect of Bacillus coagulans on Candida Metabolism

To evaluate whether the *B. coagulans*—*Candida* co-incubation could determine a long-term effect on fungal metabolic activity, 500 µL of the *C. albicans* or *C. parapsilosis* (both 1 × 10^5^ CFU/mL) in YPD broth was seeded in a 24-well plate (Sarstedt, Nümbrecht, Germany), 500 µL of *B. coagulans* (1 × 10^8^ CFU/mL) in TSB was added to each well, and incubation was carried out at 37 °C for 24 h. In control samples, sterile TSB was added. At the end of the incubation time, an aliquot of each sample was collected and seeded on SDA supplemented with chloramphenicol to exclude *B. coagulans* growth. Plates were incubated at 37 °C for 24 h. Plates were then washed to collect the grown fungi and 100 μL of both *C. albicans* and *C. parapsilosis* suspensions (1 × 10^6^ CFU/mL) was seeded in DMEM supplemented with 5% FBS in a 96-well plate and incubated at 37 °C for 24 h. Then, the medium was removed from each well, and 100 µL of 2,3-bis(2-methoxy-4-nitro-5-sulfophenyl)-2H-tetrazolium-5-carboxanilide (XTT, Sigma Aldrich, St. Louis, MO, USA) solution, supplemented with menadione (1 μM, Sigma-Aldrich, St. Louis, MO, USA), was added. The plate was covered with tinfoil and incubated at 37 °C for 3 h. Finally, 80 µL was collected from each well, transferred to another 96-well plate, and the color intensity was quantified by measuring the OD at 492 nm wavelength. 

### 2.8. Establishment of the Monolayer of Vaginal and Intestinal Epithelial Cells

Two different human epithelial cell lines were employed: the A-431 cell line from vaginal epithelial squamous cell carcinoma (ATCC CLR-1555) and the CaCo-2 cell line from colon–rectal adenocarcinoma (ATCC HTB-37). Both cell lines were cultured in DMEM (SIAL Srl, Roma, Italy) supplemented with L-glutamine (2 nM) (Euroclone SpA, Pero, Italy), penicillin (100 U/mL) (Euroclone SpA, Pero, Italy), streptomycin (100 μL/mL) (Euroclone SpA, Pero, Italy), ciprofloxacin (20 mg/mL) (Euroclone SpA, Pero, Italy), and FBS (Fetal Bovine Serum, SIAL Srl, Roma, Italy). The cell lines were kept viable by sub-culturing twice a week and keeping them at 37 °C with 5% CO_2_. Epithelial cell monolayers were obtained by seeding the cells in a 24-well plate (Sarstedt, Nümbrecht, Germany) (5 × 10^5^/mL, 1 mL/well) and by incubating the plate for 1 day (A-431) and 2 days (CaCo-2) at 37 °C with 5% CO_2_. Before being infected, the epithelial cell monolayers were washed with warmed PBS. DMEM supplemented with 10% FBS was employed for monolayer establishment, whereas DMEM supplemented with 5% FBS was used for the experiments. In the experiments including *B. coagulans*, antibiotics were not added to the medium.

### 2.9. Effect of Bacillus coagulans on C. albicans-Induced Vaginal Epithelial Cell Damage

Five hundred microliters of *B. coagulans* were seeded on a vaginal epithelial cell monolayer (bacteria/vaginal cells, Multiplicity-Of-Infection (MOI 100/1) and incubated at 37 °C with 5% CO_2_ for 6 h. Five hundred microliters of *C. albicans* were then added to each well (*C. albicans*/vaginal cells, MOI 1/1), then the plate was incubated at 37 °C with 5% CO_2_ for a further 18 h. After the incubation, lactate dehydrogenase (LDH) release in the surrounding medium was measured by a commercial kit (Hoffmann-La Roche, Basel, Switzerland). The percentage of damage was calculated following the manufacturer’s instructions. Due to LDH activity impairment caused by acidification, a bacteria-acid-producer-specific protocol was employed for samples containing *B. coagulans*, as previously described [27]. Briefly, the culture medium of each well was removed, and wells were washed with warmed HBSS (Gibco, Waltham, MA, USA). Vaginal epithelial cells were then lysed with 0.1% Triton X-100 (Sigma Aldrich, St. Louis, MO, USA) and the LDH assay was performed. Cell damage was calculated as the complementary percentage of the cell viability obtained by considering OD values from uninfected cells as 100%.

### 2.10. Impact of Bacillus coagulans on C. albicans Adhesion to Vaginal Epithelial Cells

A total of 500 µL of *B. coagulans* (bacteria/vaginal cells, MOI 100/1) and 500 µL of *C. albicans* (*C. albicans*/vaginal cells, MOI 1/1) were simultaneously added to a vaginal epithelial cell monolayer and incubated at 37 °C with 5% CO_2_ for 2 h. Cells devoid of bacteria and infected with the fungus were used as control. After the incubation, the culture medium was removed, and the wells were gently washed with warm PBS to remove non-adherent fungi. Vaginal epithelial cells were then lysed by adding 1 mL of 0.2% Triton X-100. Then, serial dilutions were performed and plated on SDA supplemented with chloramphenicol. CFU were counted after 24–48 h of incubation at 37 °C. The percentage of fungal adhesion in samples containing *C. albicans* and *B. coagulans* was calculated by considering the mean CFU values of *C. albicans* control samples as 100%. Adhesion inhibition value was calculated by subtracting the percentage of adhesion from 100.

### 2.11. β-Defensin-2 Production after C. albicans Infection in the Presence of Bacillus coagulans

The release of β-defensin-2 by vaginal epithelial cells that had been pre-colonized with *B. coagulans* for 6 h (bacteria/vaginal cells, MOI 100/1) and then infected with *C. albicans* (*C. albicans*/vaginal cells, MOI 1/1) for a further 18 h was quantified by using a commercial ELISA kit (MyBioSource, San Diego, CA, USA) according to the manufacturer’s instructions. 

### 2.12. Evaluation of Bacillus coagulans Spores’ Capacity to Germinate on the Intestinal Epithelial Cells

The CaCo-2 intestinal epithelial cell monolayer was challenged with 1 mL of DMEM-TSB (1:1) containing *B. coagulans* spores and incubated at 37 °C plus 5% CO_2_ for 24 h. Cells incubated with sterile medium were included as control. After incubation, wells were visualized by an inverted-optical microscope (Nikon Eclipse TS100, Nikon Instrument, Tokyo, Japan). Then, monolayers were lysed by adding 100 μL of 0.2% Triton X-100 to each well. After pipetting, 20 μL was deposited on a microscope glass slide and a Gram staining (Liofilchem, Roseto degli Abruzzi, TE, Italy) was performed. Slides were visualized using the Nikon Eclypse 90i (Nikon Instruments, Tokyo, Japan) microscope.

### 2.13. Statistical Analysis

All statistical analyses were carried out using GraphPad Prism 10 software. Gaussian distribution of data was confirmed by the Shapiro–Wilk test. Statistical analysis of normally distributed data was performed by using the unpaired, two-tailed Student’s *t*-test or the one-way ANOVA test followed by the uncorrected Fisher’s LSD multiple comparisons tests. Data with no Gaussian distribution were analyzed by the Kruskal–Wallis test followed by the uncorrected Dunn’s multiple comparisons test. The specific statistical analysis performed in each experiment has been detailed in the figure legends. Data in the graphs are from three different experiments. The numbers of experiments and technical replicates are specified in each figure legend. Values of * *p* < 0.05, *** *p* < 0.001, and **** *p* < 0.0001 were considered statistically significant.

## 3. Results

### 3.1. Bacillus coagulans Exerts Anti-Fungal Activity against C. albicans and C. parapsilosis

**Figure 1:**
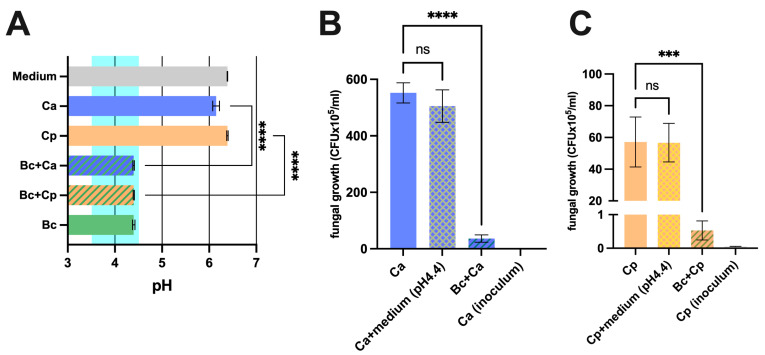
Anti-*Candida* effect exerted by *B. coagulans*. (**A**) Mono-cultures and co-cultures of *C. albicans* (Ca), *C. parapsilosis* (Cp), and *B. coagulans* (Bc) pH values after 24 h of incubation at 37 °C. The chart reports the mean values of pH ± SEM from three different experiments. The range highlighted in light green represents the mean pH levels of the healthy vaginal environment. Statistical analysis was performed by the one-way ANOVA test followed by the uncorrected Fisher’s LSD test. Ca vs. Bc + Ca **** *p* < 0.0001. Cp vs. Bc + Cp **** *p* < 0.0001. Effect of *B. coagulans* (Bc) on *C. albicans* (Ca) (**B**) and *C. parapsilosis* (Cp) (**C**) growth capacity and acidification contribution upon 24 h of incubation at 37 °C. The graph shows the mean *C. albicans* (CFU × 10^5^/mL) ± SEM from three different experiments. Statistical analysis was performed by the one-way ANOVA test followed by the uncorrected Fisher’s LSD test. Ca vs. Bc + Ca **** *p* < 0.0001. Cp vs. Bc + Cp *** *p* < 0.001. ns = not significant.

To investigate a direct antifungal effect of *Bacillus coagulans* LMG S-24828 (*B. coagulans*), the latter was co-incubated with *C. albicans* and *C. parapsilosis*, and pH values and fungal load were evaluated. The assessment of the pH values revealed that *B. coagulans* induced acidification of the medium to a final pH of 4.4. The co-incubation of *B. coagulans* with *C. albicans* or *C. parapsilosis* did not prevent such acidification. On the other hand, when *C. albicans* and *C. parapsilosis* were cultured alone, the pH of the medium returned values between 6.0 and 6.5, similar to the pH of the sterile medium (Figure 1A). Next, the antiproliferative activity of *B. coagulans* against fungal cells was evaluated by determining the fungal load in samples where *C. albicans* or *C. parapsilosis* had been incubated in the presence or absence of *B. coagulans*. In addition, to establish if the acidification per se could affect fungal growth, *C. albicans* and *C. parapsilosis* were also incubated in a sterile acidified medium (pH 4.4). The results show that *B. coagulans* significantly reduced fungal proliferation; the growth of both *Candida* species was massively affected, with a mean inhibition rate of 93.6% for *C. albicans* (Figure 1B) and 98.9% for *C. parapsilosis* (Figure 1C). On the other hand, the bare acidification of the medium did not affect the fungal growth (Figure 1B,C).

**Figure 2:**
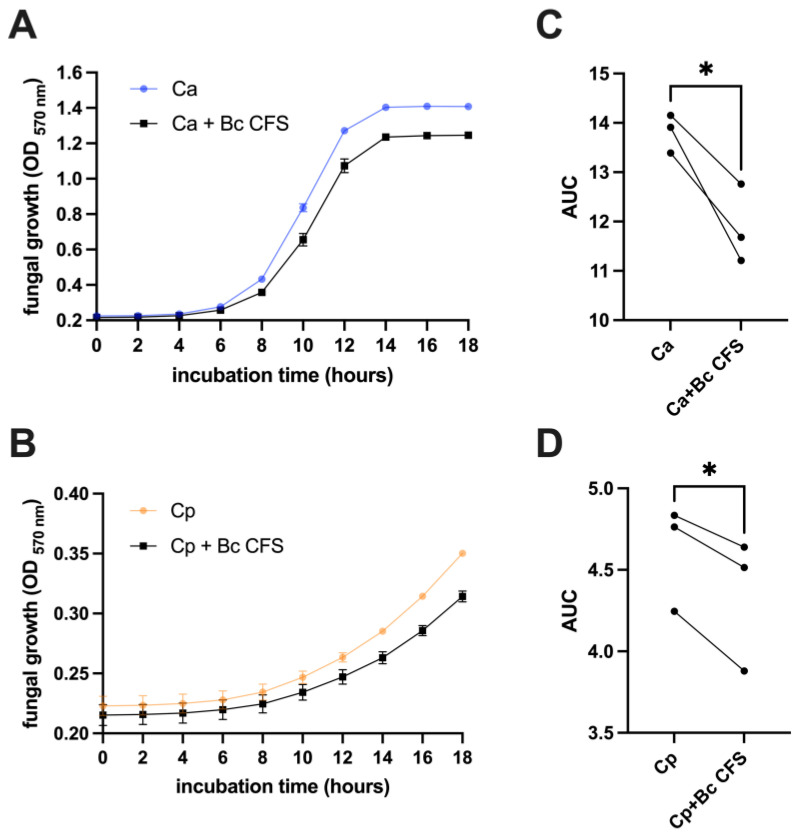
Kinetic measurement of *C. albicans* (Ca) (**A**) or *C. parapsilosis* (**B**) growth when cultivated with *B. coagulans* CFS (Bc CFS) or a sterile medium at 37 °C. Culture OD at 570 nm wavelength was automatically detected every 120 min for a total of 18 h. The graphs report the mean OD values ± SEM from triplicate samples of three different experiments. The Area Under the Curve (AUC) analysis was performed on kinetic data from *C. albicans* (**C**) and *C. parapsilosis* (**D**) samples. Each line represents one single experiment. Statistical analysis was performed on AUC values through the unpaired two-tailed Student’s *t*-test. Ca vs. Bc CFS + Ca and Cp vs. Bc CFS + Cp * *p* < 0.05.

A Cell-Free Supernatant (CFS), obtained from a 24 h *B. coagulans* culture, was employed to test if *B. coagulans* metabolites, including organic acids, could impair *C. albicans* and *C. parapsilosis* growth. Fungal cultures were grown in the presence or absence of *B. coagulans* CFS, and their growth was kinetically monitored and assessed by optical density. Finally, growth curves were drawn. For both *Candida* species, the curves show that fungal growth was reduced when the fungi were incubated with *B. coagulans* CFS (Figure 2A,B). The analysis of the area under the curves returned significantly lower OD values when both *Candida* species were grown with *B. coagulans* CFS, thus supporting the inhibitory effect of the latter (Figure 2C,D).

### 3.2. Bacillus coagulans Impairs C. albicans Adhesion to Vaginal Epithelial Cells, Reduces Hyphae Formation, and Promotes C. albicans Co-Aggregation

**Figure 3:**
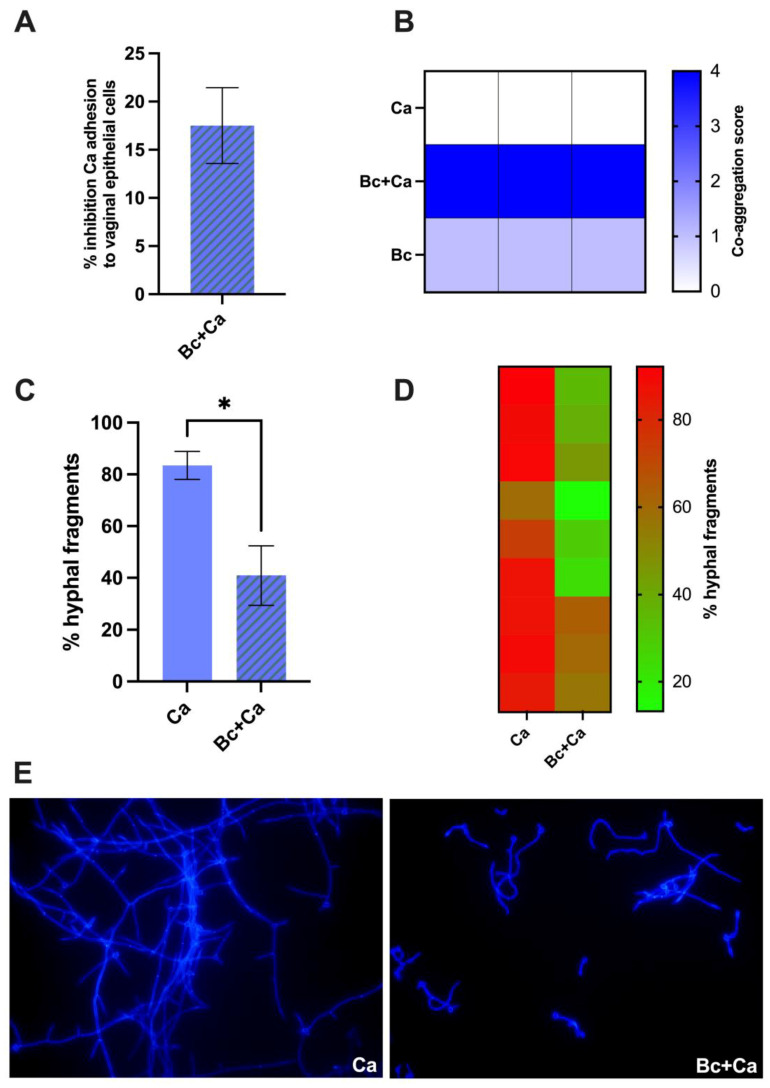
Evaluation of *C. albicans* (Ca) adhesion capacity to a vaginal epithelial cell monolayer in the presence of *B. coagulans* (Bc) (**A**). The histogram graph shows the average % ± SEM of fungal adhesion inhibition exerted by *B. coagulans* (Bc). Data are from three independent experiments. (**B**) Assessment of *B. coagulans* (Bc) capacity to co-aggregate with *C. albicans* (Ca) after 1 h of co-incubation. Boxes in the heatmap represent the score assigned to each sample in three independent experiments—0: no aggregation; 1: aggregates with small clusters; 2: aggregates with larger numbers of yeasts; 3: clumps visible with the naked eye containing large numbers of yeast cells; 4: maximum score for large clumps visible with the naked eye in the well center. (**C**–**E**) Effect of *B. coagulans* (Bc) on *C. albicans* (Ca) hyphal formation upon 4 h of co-incubation. Hyphal fragments were optically counted by fluorescent microscopy imaging. The fungal cell wall was stained with Uvitex 2B fluorescent dye. (**C**) The bars chart reports the mean percentage ± SEM of hyphal fragments counted in three different fields from three independent experiments. Statistical analysis was performed by the unpaired, two-tailed Student *t*-test. Ca vs. Bc + Ca * *p* < 0.05. (**D**) The heatmap shows the % of hyphal fragments counted in each field; the squares’ color indicates the abundance of hyphae in the field (red: high % hyphal fragments; green: low % hyphal fragments). (**E**) Representative images from fluorescence microscopy analysis are shown from *C. albicans* (Ca) or *C. albicans* plus *B. coagulans* (Bc + Ca) taken at 40× magnification.

To investigate if *B. coagulans* could impair fungal adhesion, a vaginal epithelial cell monolayer was challenged with *C. albicans* with or without *B. coagulans*. After removing the non-adherent fungi, epithelial cells were lysed, and the remaining suspensions containing only live fungal cells were serially diluted and plated on SDA supplemented with chloramphenicol, to inhibit bacterial growth. As shown in Figure 3A, we obtained an average 17.5% inhibition in the capacity of *C. albicans* to adhere to vaginal epithelial cells in the presence of *B. coagulans*, as compared to the *C. albicans* capacity to adhere to vaginal epithelial cells alone. The capacity of *B. coagulans* to aggregate fungal cells is one of the hypothesized mechanisms used by the bacterium to hinder *C. albicans* adhesion to the epithelium. By its aggregation capacity, *B. coagulans* forces *C. albicans* into closer contact with potential antifungal metabolites, therefore preventing fungal adhesion to target host sites. Here, we evaluated the aggregation between *B. coagulans* and *C. albicans* by visual assessment; after 1 h of co-incubation, a large clump, indicating coaggregation between bacteria and fungi, was visible at the center of the well, and according to the scoring system described by Pericolini E. and coworkers [19], it was assigned a score of 4. Interestingly, *B. coagulans* was also capable of self-aggregating, though less efficiently (score 2) (Figure 3B). Moreover, a hyphal formation assay was performed to investigate if *B. coagulans* could affect *C. albicans* dimorphic transition. After 4 h co-incubation of *B. coagulans* and *C. albicans*, a significant decrease in the percentage of hyphal fragments could be observed in samples where *C. albicans* was grown with *B. coagulans*, as compared to samples where *C. albicans* was grown alone (Figure 3C). Figure 3D shows a heatmap representing the percentage of hyphal fragments in each different experiment performed. Figure 3E shows representative images of the fluorescence microscopy analysis, revealing fewer and shorter hyphal fragments when the fungus was co-incubated with *B. coagulans*.

### 3.3. Beneficial Effects of Bacillus coagulans against C. albicans Vaginal Epithelial Cell Infection

**Figure 4:**
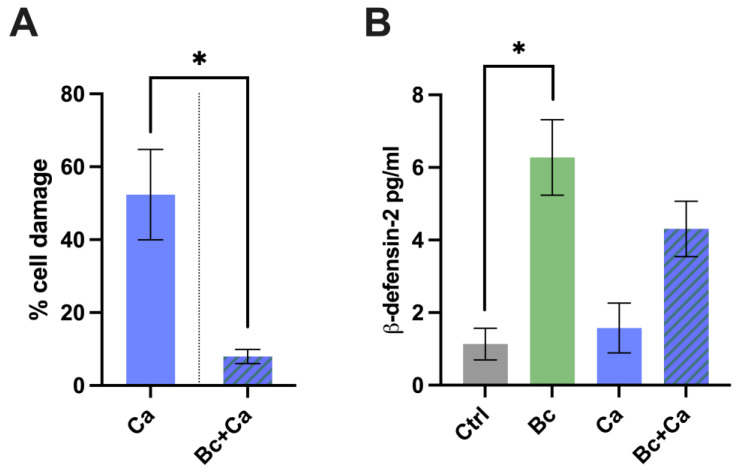
(**A**) Percentage of vaginal cell damage pre-colonized or not by *B. coagulans* (Bc) for 6 h and infected for further 18 h with *C. albicans* (Ca). The chart reports the average percentage of cell damage ± SEM of triplicate samples from three different experiments. Statistical analysis was performed by the unpaired, two-tailed Student *t*-test. Ca vs. Bc + Ca * *p* < 0.05. (**B**) Production of β-defensin-2 by vaginal epithelial cells pre-colonized or not by *B. coagulans* (Bc) for 6 h and infected for further 18 h with *C. albicans* (Ca). Uninfected cells (Ctrl) and cells colonized by the bacterium without *C. albicans* were also included in the experiments. The graph reports the mean ± SEM from three independent experiments. Statistical analysis was performed by the one-way ANOVA test followed by the uncorrected Fisher’s LSD test. Untreated cells vs. Bc pre-colonized cells * *p* < 0.05.

Next, we tested a possible protective role of *B. coagulans* against the damage induced by *C. albicans* on vaginal epithelial cells. After colonization with *B. coagulans*, the vaginal epithelial cells were infected with *C. albicans*, and the cell damage was then quantified by measurement of lactate dehydrogenase (LDH). Our results show a significant reduction in *C. albicans*-induced cell damage in *Candida*-infected vaginal epithelial cells pre-colonized with *B. coagulans*, with respect to the higher level of cell damage observed in vaginal epithelial cells that had not undergone the pre-incubation with *B. coagulans* (Figure 4A). 

In the same experimental setting, we also tested the capacity of *B. coagulans* to improve vaginal epithelial cell response to *C. albicans* by analyzing the release of the antimicrobial peptide (AMP) β-defensin-2 by vaginal epithelial cells pre-colonized with *B. coagulans*. The results show that *B. coagulans* induced per se a significant production of β-defensin-2 by vaginal epithelial cells. β-defensin-2 production was also increased in vaginal epithelial cells pre-colonized with *B. coagulans* and then challenged with *C. albicans*, thus demonstrating that *B. coagulans* potentiates the capacity of the infected epithelium to produce AMPs (Figure 4B).

### 3.4. Additional Bacillus coagulans Features for Its Potential Use in a Probiotic Formulation to Treat Candida Vaginal Infections

**Figure 5:**
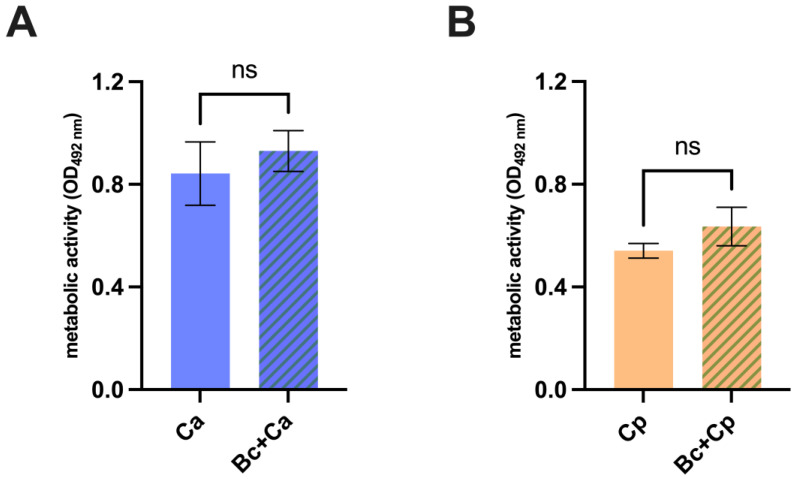

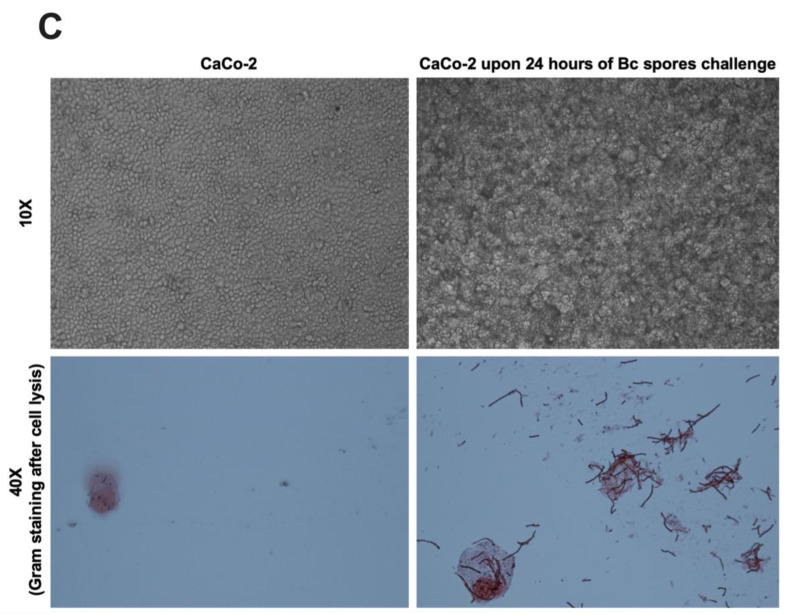
Evaluation of antifungal effect permanency upon *B. coagulans* removal. *C. albicans* (Ca) (**A**) and *C. parapsilosis* (Cp) (**B**) metabolic activity quantification after being incubated with *B. coagulans* or sterile medium for 24 h and subsequent fungal isolation and cultivation for 24 h in the lack of bacteria. The graphs show the mean OD at 492 nm wavelength ± SEM from triplicate sample of three different experiments. Statistical analysis was performed by the one-way ANOVA test followed by the uncorrected Fisher’s LSD test. ns = not significant. (**C**) Capacity of *B. coagulans* spores to germinate on intestinal epithelial cells CaCo-2. Bacterial spores were seeded on an intestinal epithelial cell monolayer of CaCo-2 and incubated at 37 °C + 5% CO_2_ for 24 h. After incubation, monolayers were photographed (upper images) and subsequently lysed. A Gram staining was then performed to visualize the presence of germinated *B. coagulans* (Bc) (lower images).

The persistence of the anti-*Candida* effects after removal of *B. coagulans* was investigated. To this end, *B. coagulans* was co-incubated with *C. albicans* or *C. parapsilosis*. Then, *B. coagulans* was removed by seeding the samples on chloramphenicol-supplemented agar medium. Finally, *Candida* was extracted and grown in fresh medium overnight and subsequently tested by XTT assay. Fungi non-precultured with *B. coagulans* and grown in sterile medium were employed as control. The results show the lack of a long-term effect on fungal metabolic activity in both *C. albicans* (Figure 5A) and *C. parapsilosis* (Figure 5B) after *B. coagulans* removal. Indeed, no differences in metabolic activity could be observed by comparing the two different *Candida* cultures. Last, we evaluated the capacity of *B. coagulans* spores to germinate on human intestinal epithelial cells (i.e., CaCo-2 cell line). The results show that productive germination occurred after 24 h from the spore’s inoculum. The Gram staining confirmed the presence of Gram-positive bacilli, which were absent in the untreated CaCo-2, used as negative control (Figure 5C).

## 4. Discussion

Probiotics are living microbes that confer a health benefit to the human host. The anti-pathogen activity, the immunomodulatory effect, and the tolerated proliferation within the human organism are some of the acknowledged key features of probiotics. Research on probiotics has rapidly increased over the last few decades, and probiotic administration is considered a promising therapeutic approach to treat various diseases. Yet very little information is available to date on the efficacy of probiotics in the treatment and prevention of VVC. In the present study, we have investigated the probiotic potentiality of *Bacillus coagulans* (*B. coagulans*) LMG S-24828 (Weizy^®^) in the context of *Candida*-associated vaginal infections by employing a vaginal epithelial cell infection model. We have started by evaluating the capacity of *B. coagulans* to affect *Candida* virulence traits related to invasiveness and pathogenicity, such as fungal growth, adhesion to epithelial cells, and dimorphic transition. A healthy vaginal environment is characterized by an acidic pH with values around 4. The acidification is granted by resident bacteria, such as *Lactobacillus* spp., that produce lactic acid as the end-product of their fermentative metabolism. In addition, several other species included in commercial probiotic formulations contribute to the acidification of the vaginal environment, thus maintaining or restoring eubiotic conditions. Recently, it has been demonstrated that *B. coagulans* too is able to produce lactic acid [28,29]. Indeed, here we show that *B. coagulans* induces acidification to pH values similar to those occurring in an eubiotic vaginal milieu. Interestingly, such acidification capacity is not hindered by co-culturing *B. coagulans* with *C. albicans* or *C. parapsilosis*. Such an ability to provide an acidic environment is a necessary feature (albeit not sufficient per se) of every probiotic formulation intended for the recovery of vaginal eubiosis, which explains the importance of this result.

*Candida* can physiologically colonize a healthy vaginal mucosa without inducing symptoms, thanks to a constant inhibition of its overgrowth granted by the immune system and by the resident microbiota [30]. Therefore, the antiproliferative activity against *Candida* is another pivotal attribute of probiotic formulations intended to treat or prevent vaginal infections. Our results show that *B. coagulans* massively affects *Candida* growth, and this effect is not due only to the mere acidification of the medium, since both *C. albicans* and *C. parapsilosis* have been able to grow on *B. coagulans*-free medium, acidified to a pH of 4.4. Interestingly, the evaluation of anti-proliferative activity mediated by Cell-Free Supernatant (CFS) obtained from *B. coagulans* culture showed that even metabolites produced by this bacterial strain can reduce fungal growth. Therefore, it is possible that the acidification induced by *B. coagulans* per se may affect *Candida*, but that other metabolites and/or yet unraveled mechanisms must be involved to obtain an effective impairment of fungal growth. This would explain why *Candida* growth is not inhibited in a *B. coagulans*-free acidified medium. A similar effect has also been described for *C. parapsilosis* [30]. Besides supporting the probiotic features of *B. coagulans* LMG S-24828, these findings also suggest its possible postbiotic characteristics.

The capacity to adhere to cells is the first step for *Candida* to begin the infection process. Here, we show that *B. coagulans* impairs *C. albicans* adhesion and this seems to be due mostly to its capacity to co-aggregate *Candida*. Indeed, co-aggregation is an important mechanism used by probiotics to clear microbial pathogens: through co-aggregation, the probiotics can create a competitive microenvironment that compromises the capacity of the pathogens to adhere to the epithelial cells. This is, ultimately, an effective means for preventing *Candida* adhesion and infection [19].

As a dimorphic fungus, *C. albicans* can exist in two different morphologies: yeast cells or hyphal forms. When *C. albicans* dwells in the acidic vaginal niche of healthy women as a harmless colonizer, the yeast form is prevalent and, in this form, *Candida* is well tolerated by the genital mucosa. On the other hand, the hyphal form becomes prevalent during symptomatic VVC episodes. The elongation of hyphal structures causes direct damage to epithelial cells, through active penetration driven by the mechanical force of the elongating hypha; this, in turn, induces neutrophil recruitment and triggers a wide inflammatory response due to the high levels of cellular damage [31]. Moreover, the presence of hyphae is linked to the production of candidalysin, the only toxin demonstrated to be produced by *C. albicans* and only in its hyphal form. This toxin exerts cytolytic activity and induces cell damage through the induction of mtROS by vaginal epithelial cells [32,33]. Here, we demonstrate that the co-culture of *C. albicans* with *B. coagulans* dampens the hyphal elongation capacity, as shown by the observation of shorter and fewer hyphal fragments by fluorescence microscopy. Under such an impairment of the *C. albicans* dimorphic transition, an important role may be played by the acidification induced by *B. coagulans*. Indeed, several studies report that acidic pH values inhibit *C. albicans* switching from yeast to hyphal form [34,35]

To contextualize the observed anti-*Candida* effects of *B. coagulans* in the VVC scenario, we have employed an in vitro infection model consisting of a vaginal epithelial cell monolayer infected with *C. albicans* in the presence or absence of *B. coagulans*. With this model, we have demonstrated that upon fungal challenge, the presence of the *Bacillus* correlates with a reduction in fungal-induced epithelial cell damage. In addition, we have assessed the production of AMPs by vaginal epithelial cells, in order to establish if *B. coagulans* is capable of alerting the epithelium to an impending danger and stimulating a response. Indeed, our data show that following pre-colonization with *B. coagulans*, the epithelial cells are activated and increase the release of the alarmin β-defensin-2, a molecule with a potent antimicrobial activity against Gram-negative bacteria and *Candida* [36,37].

VVC cases due to non-*albicans Candida* species have always been uncommon, but they are progressively increasing. Non-*albicans Candida* species have been demonstrated to become resistant to antifungals at a higher rate than *C. albicans* [38]; hence, preventing a *Candida* infection through the application of probiotics may provide beneficial effects, especially against drug-resistant species. For this reason, notwithstanding that the main goal of this project is the demonstration of the beneficial effects of *B. coagulans* LMG S-24828 against *C. albicans*, a *C. parapsilosis* reference strain was also included in some of the experiments described above. Interestingly, the effects of *B. coagulans* on *C. parapsilosis* are superimposable to the effects on *C. albicans*. Indeed, the hindering of both *C. albicans* and *C. parapsilosis* growth to a similar extent suggests that the effects of *B. coagulans* LMG S-24828 are not species-specific, and they might affect a broad spectrum of *Candida* species. 

A complex crosstalk links the gut to the vaginal microbiota, involving a continuous bacterial species translocation through these body sites, thanks to their anatomical proximity. Indeed, the origin of vagina colonizer bacteria, such as *Lactobacillus* spp., has been traced back to the rectum, which also acts as a reservoir of beneficial microbes. In addition to a direct migration from the rectum, bacteria translocation has also been demonstrated to occur through hematogenous transfer from the gut to the uterus [12]. Such crosstalk is an essential feature, because most probiotic formulations are typically consumed by ingestion, and the proliferation of beneficial bacteria, as well as the germination of the spores, occur on the surface of intestinal mucosa. For spore-based probiotic formulations in particular, efficient germination on intestinal epithelial cells is essential in targeting beneficial bacteria to the gut. Our results show that the spores of *B. coagulans* LMG S-24828 can germinate on the intestinal epithelial cell line CaCo-2, which adds up to the data that indicate this strain as suitable to be employed as an effective probiotic for the prevention and treatment of vaginal epithelial infections. All the data presented and discussed here have been generated by an in vitro model. Further studies will be warranted to also assess the anti-fungal effects of the *B. coagulans* LMG S-24828 strain in an in vivo setting. In addition, according to the results obtained by employing the *Bacillus* CFS, it will be interesting to define and characterize those *Bacillus* metabolites that may act as postbiotics.

## 5. Conclusions

**Figure 6:**
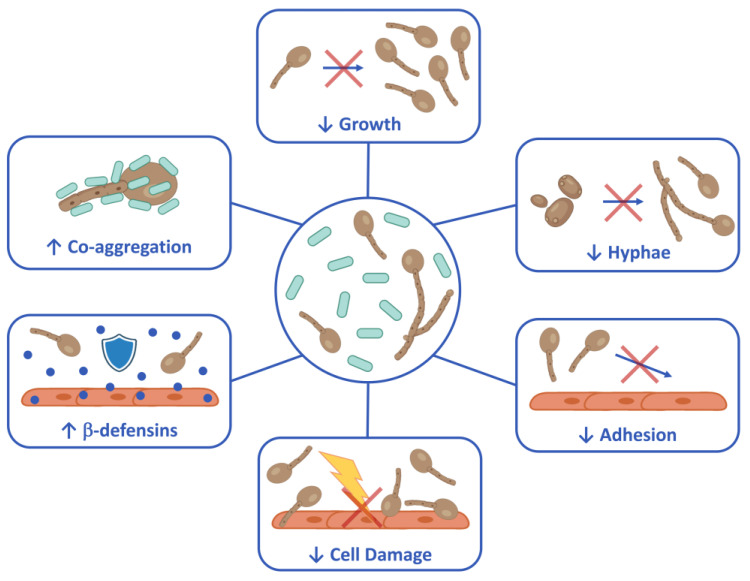
Schematic representation of biological activities of *B. coagulans* LMG S-24828 against *Candida*. Created with BioRender.com.

The spore-forming *B. coagulans* LMG S-24828 has shown a marked antifungal activity against *C. albicans* and *C. parapsilosis*, resulting in a protective effect toward vaginal epithelial cells infected by fungi. Specifically, as summarized in Figure 6, the inhibition of fungal growth both by the bacteria and by their secreted molecules, the inhibition of hyphae formation, the reduction in fungal adhesion, the capacity to co-aggregate with *C. albicans*, as well as the effects on the epithelial cells (reduction in *Candida*-induced cell damage and increase in β-defensin-2 secretion) are all features exhibited by *B. coagulans* in this in vitro study. Taken together, the in vitro data presented here suggest that *B. coagulans* LMG S-24828 has beneficial effects, which makes this bacterium a potential therapeutic tool for the prevention and treatment of *Candida* vaginal infections. Further studies are warranted to better characterize its probiotic and postbiotic activities in vivo. Moreover, a clinical trial should be performed to fully demonstrate its capacity to colonize the intestine and the vaginal cavity, providing benefits against VVC. 

## Data Availability

The original contributions presented in the study are included in the article, further inquiries can be directed to the corresponding authors.
